# Application and efficacy of the pubovesical complex-preserving technique in intrafascial laparoscopic radical prostatectomy: A propensity score-matched analysis

**DOI:** 10.1371/journal.pone.0342248

**Published:** 2026-03-06

**Authors:** Zaisheng Zhu, Yiyi Zhu, Han Wu, Pengfei Zhou, Quanqi Liu, Jianyong Tong, Yueping Wang

**Affiliations:** 1 Department of Urology, Jinhua Hospital Affiliated to Zhejiang University School of Medicine, Jinhua, China; 2 Department of Endocrinology, Affiliated Second Hospital to Zhejiang University School of Medicine, Hangzhou, China; Jan Biziel University Hospital No 2 in Bydgoszcz: Szpital Uniwersytecki Nr 2 im dr Jana Biziela w Bydgoszczy, POLAND

## Abstract

**Purpose:**

This study aimed to introduce the pubovesical complex preserving technique in intrafascial laparoscopic radical prostatectomy (PPLRP) and compare its efficacy with the conventional intrafascial laparoscopic radical prostatectomy (Intra-LRP).

**Materials and methods:**

The clinical data of 475 cases who underwent PPLRP (n = 171) or Intra-LRP (n = 304) between 2012 and 2023 and were followed up for over 1 year were retrospectively analyzed. The baseline characteristics of the two groups were balanced using 1:1 propensity score matching. Lastly, Kaplan-Meier analysis and multivariable logistic regression were performed to assess survival rates and their influencing factors.

**Results:**

After matching, 310 cases (155 cases each) were successfully matched. The median follow-up for the two groups was 45 and 47 months, respectively. The rate of positive surgical margins (PSM) was comparable between the two groups at 17.4% and 14.8% (p = 0.537), respectively. Moreover, Kaplan-Meier analysis revealed no significant difference in the biochemical relapse-free survival (bRFS) (p = 0.772), cancer-specific survival (CSS) (p = 0.507), and overall survival (OS)(p = 0.065) between the two groups. PPLRP was associated with earlier recovery of urinary control, as evidenced by higher continence rates 24 hours following catheter removal (p = 0.002), as well as at 1 month (p = 0.011), 3 months (p < 0.001), and 6 months (p = 0.020). Regarding sexual function, postoperative IIEF-5 scores were significantly higher in the PPLRP group compared to the intra-LRP group (p < 0.001), and the proportion of patients achieving satisfactory erections for sexual intercourse was 31.0% and 19.4%, respectively (p = 0.018). Finally, multivariate analysis identified surgical technique as the sole factor affecting urinary continence recovery (p = 0.001), while surgical technique (p = 0.042) and age (p = 0.050) were associated with sexual function recovery.

**Conclusion:**

Compared with traditional Intra-LRP, PPLRP offered superior preservation of the pubovesical complex (PVC) and neurovascular bundle (NVB). Indeed, it facilitated early recovery of urinary continence and sexual function without increasing oncological risk. Besides, it can be feasibly implemented in hospitals equipped with conventional laparoscopic systems.

## Introduction

As is well documented, surgical treatments for prostate cancer include open, laparoscopic, and robotic-assisted laparoscopic radical prostatectomy (RALP). In recent years, RALP has become increasingly popular, especially in developed countries such as the USA [1,2]. Due to the high costs associated with robotic systems and consumables, open and laparoscopic radical prostatectomy (LRP) remain the most commonly used surgical methods worldwide, especially in developing countries [3,4]. While RALP allows for precise dissection via three-dimensional visualization with 8-12x magnification and early recovery of urinary continence and erectile function, its long-term benefits remain elusive [3–7], and its cost-effectiveness remains suboptimal [3,8]. Surgical technique is the only variable factor that affects early functional recovery postoperatively [9]. Several novel improvements to RALP have been reported [2,9–13]. Among them, two have been widely applied, namely Retzius-sparing prostatectomy [9–11] and the “veil of Aphrodite” [2,12,13] technique. To date, studies investigating the implementation of these new techniques in open/standard laparoscopy are scarce. The posterior approach is characterized by a narrow operative field and is relatively challenging to perform under standard laparoscopy, whereas the anterior approach (posterior to the symphysis pubis) is similar to open and standard laparoscopy. Since 2011, the “veil-sparing” technique has been implemented in open [14] and conventional laparoscopy, and technical improvements have been made for the preservation of the pubovesical complex (PVC) (i.e., pubovesical complex-preserving technique for intracial laparoscopic radical prostatectomy, PPLRP [15]). Notably, this technique fully preserves anatomical structures such as the puboprostatic ligament, dorsal vein complex (DVC), detrusor apron and neurovascular bundle (NVB). When combined with bilateral intrafascial nerve-sparing, it is completely identical to the currently reported robot-assisted super-veil-sparing technique [16] and HOOD technique [17]. Herein, the PPLRP technique was introduced and compared with the conventional laparoscopic intrafascial nerve-sparing (intrafascial laparoscopic radical prostatectomy, Intra-LRP) technique in terms of oncological outcomes, early functional recovery, and complications.

## Materials and methods

Between July 2012 and December 2023, 198 PPLRP and 493 Intra-LRP procedures were performed in our institution. Multidisciplinary discussion was held preoperatively for both groups, with consensus reached on the applicability of nerve-sparing surgery. All interventions were conducted by a high-volume surgeon (ZHU). A total of 136 cases were excluded from the study (33 patients lacking follow-up data beyond 1 year and 106 patients who underwent immediate endocrine therapy postoperatively). Ultimately, 475 cases were included in the retrospective analysis (PPLRP, n = 171 and Intra-LRP, n = 304). Propensity score matching was performed to minimize confounding factors such as selection bias between groups, and efficacy outcomes, including postoperative oncological conditions, urinary control, sexual function recovery, and complication rates, were compared between the two groups.

### Propensity score matching

To control for confounding factors and selection bias between groups, propensity score matching was performed at a 1:1 ratio based on preoperative clinical variables using a multivariable logistic regression model. Variables matched comprised age, body mass index (BMI), comorbidities, preoperative IIEF-5 score, ASA score, postate volume, PSA level, Gleason grade, clinical stage (based on the TNM staging system [18]), NCCN risk classification, nerve-sparing status (bilateral/unilateral), pelvic lymph node dissection (PLND), receipt of postoperative adjuvant radiotherapy, and follow-up duration. The propensity score was used to standardize covariates and balance baseline differences between groups.

Tumor grade grouping was determined based on the 2014 grading system established by the International Society of Urological Pathology [18] and graded according to the NCCN-recommended prostate cancer prognostic risk stratification (D-Amico scale) [19].

### Surgical technique

**Fig 1:**
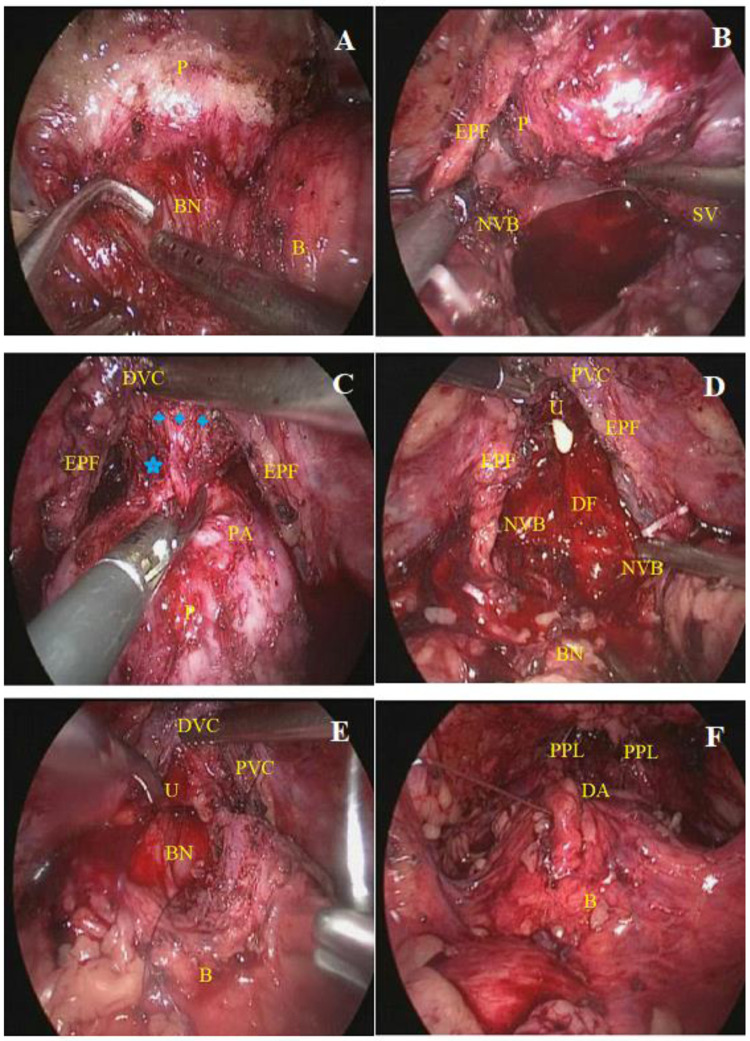
Intraoperative view of PPLRP (bilateral nerve-sparing). **A:** Complete preservation of the bladder neck. **B:** Light intrafascial dissection:Posterior prostate dissection in the intrafascial plane and prostatic pedicle(including NVB) dissection. **C:** Dissection of the apex and detachment of the DVC from the prostate by cold cutting with scissors(Blue pentagram: Membranous urethra;Blue four-pointed star: Fibromuscular tissue between the DVC and the prostate.).**D:** Wide preservation of the PVC complex: including the puboprostatic ligament, DVC,detrusor apron, pelvic fascia, and NVB. **E:** An assistant lifted the widely-preserved PVC, beneficial for the anastomosis of bladder neck and urethra. **F:** Anatomical reduction PVC. PPLRP, pubovesical complex preserving technique in intrafascial laparoscopic radical prostatectomy. **The labeling in Fig 1 is as follows:** B = bladder, BN = Bladder neck, DA = detrusor apron, DF = Denonvilliers’fascia, DVC = dorsal vein complex, EPF = endopelvic fascia, NVB = neurovascular bundle, P = prostate, PA = prostate apex, PPL = Puboprostatic ligament,PVC = Pubovesical complex, SV = seminal vesicle,U = urethra.

The PPLRP technique [15] was performed via an anterior approach. Firstly, the subcutaneous fat tissue over the prostate surface was removed, following which the bladder neck was transversely incised between the 10–2 o’clock positions at the junction of the bladder neck and prostate. Next, the bladder was retracted cephalad to expose and transect the bladder-urethral muscle fibers located at the junction, thereby preserving the bladder neck (Fig 1A). The anterior and posterior lips of the bladder neck were divided using a harmonic scalpel or cold scissors. Afterward, the epididymis and vas deferens were exposed, and the Denonvillier’s fascia was incised sharply or bluntly close to the epididymis. Then, the dissection was extended bilaterally to release the NVB and free the pelvic side fascia (Fig 1B). Thereafter, the apex was dissected, and the DVC was detached from the prostate. The fibromuscular tissue between the DVC and the prostate was dissected using cold scissors along the midline, and the urethra was transected (Fig 1C). At this stage, the entire PVC [including all structures surrounding the membranous urethra around the prostate (3600), such as the puboprostatic ligament, DVC, detrusor apron, pelvic fascia, and NVB, etc.] was completely preserved (Fig 1D). Following this, the posterior Denonvilliers’ fascia was repaired. Anastomosis of the bladder neck and the membranous urethra was performed (Fig 1E), followed by anatomical reduction of the PVC and closure of the ventral bladder neck incision (Fig 1F). Intra-LRP adopted traditional classical techniques [2,20]. For patients with intermediate-high risk, according to the D’Amico classification and European guidelines [18,21], pelvic lymph node dissection [22] was performed.

S1 Fig shows more detailed surgical steps.Intraoperative detailed anatomy, reconstruction, and operative techniques of PPLRP procedure are more clearly described in the conventional laparoscopic approach (S1 Fig). Meanwhile, with the widespread use of high-definition conventional laparoscopy,under 4K high-definition conventional laparoscopy, perform clear and precise key technical operations such as preservation of the pudendal artery, bilateral nerves or unilateral nerve (S1 Fig).

### Follow-up and study endpoints

Oncological follow-up: Serum prostate-specific antigen (PSA) monitoring was initiated 1 month post-surgery, and serum PSA levels were monitored monthly for the first 3 months, followed by every 3 months thereafter. In cases with stable PSA levels, follow-up intervals were extended to every 6 months after 2 years. The primary oncological endpoints were biochemical relapse-free survival (bRFS), cancer-specific survival (CSS), and overall survival (OS). Biochemical recurrence was defined as a postoperative PSA level >0.2 ng/mL on two consecutive measurements [23].

Functional and complication assessment included urinary control, sexual function, and perioperative complications. All functional follow-up assessments were conducted by trainees and the nursing team during follow-up visits at the hospital or via telephone interviews. Urinary control was evaluated by inquiring about the number of pads used by the patient each day at specific time points, immediately (within 24 hours after catheter removal), and at 1, 3, 6, and 12 months postoperatively, allowing a follow-up window of ±1 week. [12] Incontinence was defined as the use of ≥1 pads daily, whereas complete urinary control was defined as the absence of pad use. Sexual function was assessed by inquiring whether the patient’s sexual function has recovered to the preoperative level. Sexual function assessment included preoperative fertility status, sexual activity status (masturbation, foreplay, and sexual intercourse), and erectile function [24,25]. Erectile function was assessed using the IIEF-5 scoring questionnaire [5,24]. Follow-up was initiated at 6 months postoperatively, with evaluation focused on the best sexual function performance reported. Recovery was defined as the patient’s subjective perception that sexual function had returned to the preoperative level or the ability to achieve satisfactory erectile rigidity and engage in sexual intercourse regardless of medication use (such as phosphodiesterase type 5 (PDE5) inhibitors) [25,26]. Complications were recorded using the Clavien-Dindo system [27].

This study was conducted in compliance with the principles of the Declaration of Helsinki.This study was approved by the Ethics Review Committee of Jinhua Central Hospital (approval number: 2021−187). All patients have signed informed consent forms. All methods were performed following the relevant guidelines and regulations. Ethical review approval document (S1 Appendix).

### Statistical analysis

SPSS (27.0 IBM) was employed for propensity score matching and statistical analyses. Baseline analysis was performed on variables affecting the study endpoints. Continuous variables were compared using the Student’s t-test or Mann-Whitney U test, whereas categorical variables were compared using the χ2 test or Fisher’s exact test. Kaplan-Meier survival analysis was performed to assess postoperative bRFS, CSS and OS, with statistical significance evaluated using the log-rank test. A Logistic regression model was established to identify factors affecting urinary control and sexual function. p < 0.05 was considered statistically significant.

## Results

Participants were assigned to either the PPLRP group (n = 171) or the intra-LRP group (n = 304). PSM matching was successfully performed for 310 patients (155 in each group). Post-matching evaluation confirmed balanced clinical characteristics between the two groups, with a matching ratio of 1:1 and a tolerance level of 0.02. (Table 1).

**Table 1:** Comparison of clinical characteristics between PPLRP and Intra-LRP groups before and after propensity score matching.

Variables[Mean ± SD, or n (%)]	Before propensity score matching			After propensity score matching		
	PPLRP (n = 171)	Intra-LRP (n = 304)	p-value	PPLRP (n = 155)	Intra-LRP (n = 155)	p-value
Age (years)	66.18 ± 6.24	69.05 ± 5.94	<0.001	66.48 ± 6.27	67.07 ± 5.98	0.399
BMI (kg/m^2^)	22.69 ± 2.84	22.94 ± 2.92	0.359	22.66 ± 2.86	22.50 ± 2.97	0.633
Associated comorbidities			0.255			0.858
Hypertension	42 (24.6)	82 (27.0)		38 (24.5)	33 (21.3)	
Diabetes	11(6.4)	28 (92.0)		10 (6.5)	13 (8.4)	
Heart disease	5(2.9)	17 (5.6)		5 (3.2)	5 (3.2)	
PSA (ng/mL)	15.15 ± 12.08	16.75 ± 13.63	0.201	15.56 ± 12.37	17.03 ± 14.24	0.330
Prostate volume (mL)	38.34 ± 17.32	40.01 ± 17.42	0.315	38.55 ± 17.78	39.33 ± 17.18	0.693
ASA Physical Status			0.636			0.123
≤2	150 (87.7)	262 (86.2)		135 (87.1)	125 (80.6)	
>3	21 (25.9)	42 (13.8)		20(12.9)	30(19.4)	
Biopsy Gleason grading grouping			0.955			0.076
1	59(34.5)	109 (35.9)		51 (32.9)	55 (35.5)	
2-3	95 (55.6)	166 (54.6)		89 (57.4)	73 (47.1)	
4-5	17 (9.9)	29 (9.50)		15 (9.70)	27 (17.4)	
Clinical T staging			0.732			0.658
cT1-T2a	88 (51.5)	145 (47.7)		80 (51.6)	79 (51.0)	
cT2b	53 (31.0)	104 (33.6)		45 (29.0)	51 (32.9)	
cT2c	30 (17.5)	57 (18.8)		30 (19.4)	25 (16.1)	
NCCN risk classification			0.340			0.323
low risk	26 (15.2)	35 (11.5)		21 (13.5)	23 (14.8)	
Intermediate risk	86 (50.2)	147 (48.4)		77 (49.7)	64 (41.3)	
high risk	59 (34.5)	122 (40.1)		57 (36.8)	68 (43.9)	
Nerve-Sparing			0.011			0.401
Bilateral	112 (65.5)	232 (76.3)		106 (68.4)	99 (63.9)	
Unilateral	59 (34.5)	72 (23.7)		49 (31.6)	56 (36.1)	
PLND	69 (40.4)	138 (45.4)	0.287	67(43.27)	83(53.5)	0.069
Adjuvant therapy	24 (14.0)	42 (13.8)	0.947	24(15.5)	30 (19.4)	0.369
Preoperative IIEF-5 score	18.20 ± 6.35	14.82 ± 6.45	<0.001	17.67 ± 6.40	17.65 ± 6.00	0.978
Follow-up time [M(IQR),months]	46(32.5- 72.5)	42 (29-62)	0.012**	45 (32- 66)	47 (32.5-65)	0.807**

ASA, American Society of Anesthesiologists; BMI, body mass index; IIEF-5, Index of Erectile Function-5; Intra-LRP, intrafascial laparoscopic radical prostatectomy; IQR, interquartile range; NCCN, National Comprehensive Cancer Network; M, median; PLND, pelvic lymph node dissection; PPLRP, pubovesical complex preserving technique for intrafascial laparoscopic radical prostatectomy; PSA, prostate-specific antigen; SD, standard deviation; ** Mann-Whitney U-test.

### Perioperative characteristics

Surgical duration, intraoperative blood loss volume, and perioperative morbidity rates were comparable between the two groups (p = 0.557, 0.546, and 0.821, respectively). The majority of complications [27] were classified as low grade (Clavien I–II), with only one patient in the intra-LRP group experiencing grade III complications, which was an anastomotic stricture (Table 2).

**Table 2:** Comparison of perioperative and pathological outcomes between PPLRP and Intra-LRP groups after matching.

Variables[Mean ± SD,n (%)]	PPLRP (n = 155)	Intra-LRP (n = 155)	p-value
Operation time(min)	197.71 ± 45.99	200.82 ± 47.17	0.557
Estimated blood loss(mL)	251.16 ± 160.29	257.94 ± 160.97	0.546
Postoperative complication (Clavien-Dindo grade)	10 (6.50)	11 (7.10)	0.658
I	6 (3.9)	6 (3.9)	
II	4 (2.6)	4 (2.6)	
III	0 (0.0)	1 (0.6)	
Pathological stage			0.146*
pT1-pT2	139 (89.6)	130 (84.6)	
pT3	16 (10.3)	24 (15.5)	
pT4	0 (0.00)	1 (0.60)	
Pathological Gleason grading grouping			0.064
1	38 (24.5)	49 (31.6)	
2-3	100 (64.5)	80 (51.6)	
4-5	17 (11.0)	26 (16.8)	
Positive surgical margin	27(17.4)	23 (14.8)	0.537
pT1-pT2	20 (12.9)	14 (9.0)	
pT3	7 (4.5)	8 (5.2)	
pT4	0 (0.0)	1 (0.6)	
PSM site			
Apical	10 (6.5)	8 (5.2)	0.627
Lateral	4 (2.6)	3 (1.9)	NA
Base	2 (1.3)	2 (1.3)	NA
Posterolateral	11(7.1)	10(6.5)	0.821

Intra-LRP, intrafascial laparoscopic radical prostatectomy; PPLRP, pubovesical complex preserving technique for intrafascial laparoscopic radical prostatectomy; PSM, positive surgical margin; NA, not available; SD, standard deviation; *Fisher’s exact test.

### Pathological and oncological outcomes

No significant differences were noted in pathologic characteristics such as postoperative pathologic stage and Gleason grade between the two groups (p = 0.146 and 0.064, respectively). Likewise, the rate of positive surgical margins (PSM) was comparable between the PPLRP and intra-LRP groups (17.4% vs 14.8%, p = 0.537). Interestingly, the anatomical locations of PSMs were also similar (p > 0.05), including the positive margin rate at the apex (6.5% vs 5.2%, p = 0.627) and posterolateral margins (7.1% vs 6.5%, p = 0.821) (Table 2).

**Fig 2:**
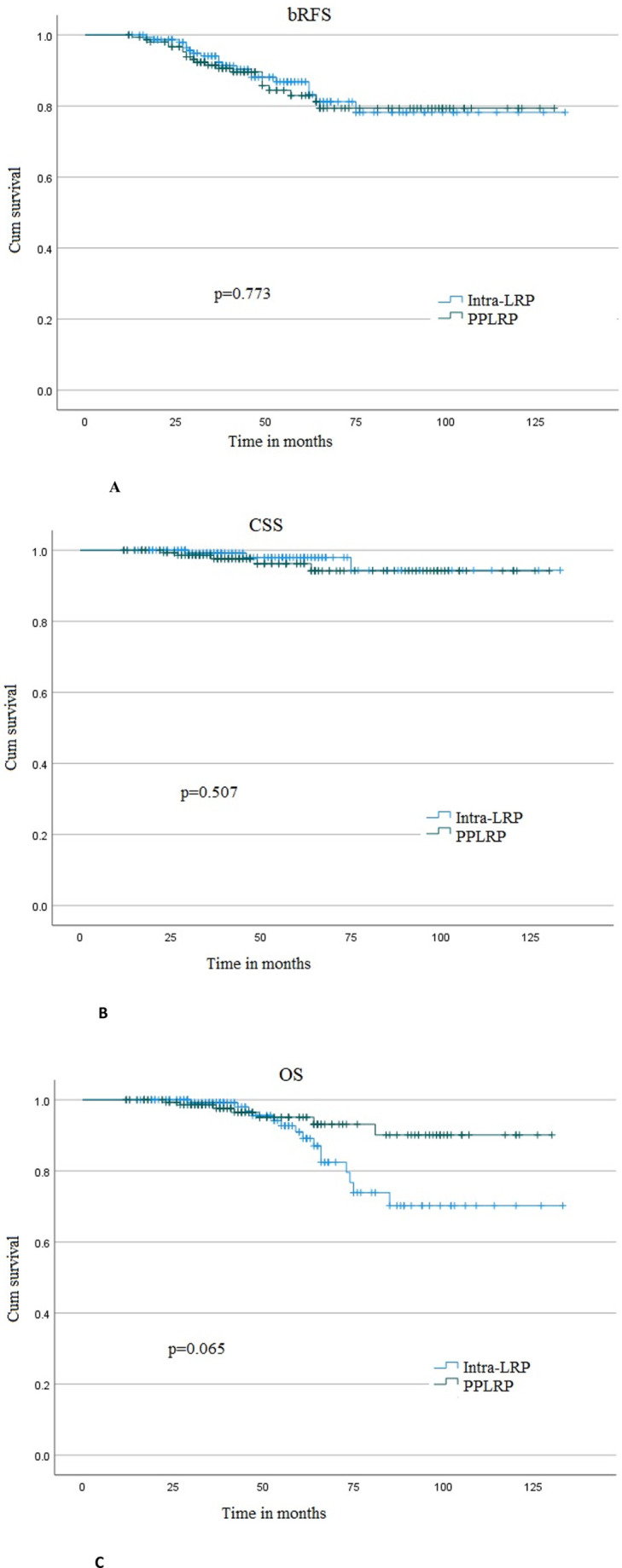
Kaplan-Meier curves. **A:** bRFS, Biochemical recurrence-free survival; **B:** CSS, Caner-specific survival; **C:** OS, Overall survival. Intra-LRP, intrafascial laparoscopic radical prostatectomy; PPLRP, pubovesical complex preserving technique in intrafascial laparoscopic radical prostatectomy.

The median follow-up duration in the PPLRP and intra-LRP groups was 45 (32–66) and 47 (32.5–65) months, respectively. Kaplan-Meier analysis revealed a bRFS rate of 82.9% and 79.4% at 5 years and 10 years in the PPLRP group, respectively, and 86.8% and 78.2% in the intra-LRP group (p = 0.773; Fig 2A). Moreover, the 5- and 10-year CSS rate was 96.2% and 94.2% in the PPLRP group, and 98.0% and 94.3% in the intra-LRP group (p = 0.507; Fig 2B). Similarly, the 5- and 10-year OS rate was 93.1% and 90.1% in the PPLRP group, and 90.9% and 70.2% in the intra-LRP group (p = 0.065; Fig 2C). Indeed, no statistically significant differences were noted between the two groups across all oncological endpoints (p > 0.05).

### Functional outcomes

The continence rates in the PPLRP and intra-LRP groups were 78.1% and 61.9% (p = 0.002) on postoperative day 1 (24 h after catheter removal), 94.8% and 86.5% (p = 0.011) at 1 month, 97.4% and 86.5% (p < 0.001) at 3 months, and 99.4% and 94.2% (p = 0.020) at 6 months, respectively. More importantly, the rate of urinary continence within 6 months postoperatively was significantly higher in the PPLRP group compared to the intra-LRP group (p < 0.05; Table 3). However, no significant difference was observed in the rate of urinary continence at 12 months between the 2 groups (p = 0.214; Table 3). Multivariable analysis revealed that surgical technique (p = 0.001) was the sole factor influencing urinary incontinence recovery (Table 4).

**Table 3:** Comparison of functional outcomes between PPLRP and Intra-LRP groups after matching.

Variables [Mean ± SD,n (%)]	PPLRP (n = 155)	Intra-LRP (n = 155)	p-value
Urination control function recovery			
24 hours	121 (78.1)	96 (61.9)	0.002
1 months	147 (94.8)	134 (86.5)	0.011
3 months	151 (97.4)	134 (86.5)	<0.001
6 months	154 (99.4)	146 (94.2)	0.020*
12 months	154 (99.4)	150 (96.8)	0.214*
Sexual functional recovery			
Postoperative IIEF-5 score	13.89 ± 3.74	10.43 ± 3.60	<0.001
Subjective perception of sexual function recovery to preoperative level	67 (43.2)	51 (32.9)	0.061
Sexual intercourse	48 (31.0)	30 (19.4)	0.018
Sexual activity except sexual intercourse	21 (13.5)	21 (13.5)	1.000

IIEF-5, Index of Erectile Function-5; Intra-LRP, intrafascial laparoscopic radical prostatectomy; PPLRP, pubovesical complex preserving technique for intrafascial laparoscopic radical prostatectomy; SD, standard deviation; *Fisher’s exact test.

**Table 4:** Univariate and multivariate analysis of sexual and urination control functional recovery after matching.

Variables	univariate analysis		multivariate analysis	
	OR (95% CI)	p-value	OR (95% CI)	p-value
Sexual functional recovery				
Surgical technique (PPLRP/Intra-LRP)	0.535 (0.317-0.904)	0.019	0.558 (0.318-0.980)	0.042
Age (years)	0.894 (0.855-0.935)	<0.001	0.940 (0.883-1.000)	0.050
PSA (ng/mL)	0.972 (0.949-0.995)	0.019		
Preoperative IIEF-5 score	1.121 (1.006-1.180)	<0.001		
NCCN risk classification		0.044		
low risk	1			
Intermediate risk	1.963(0.887-4.346)	0.096		
high risk	2.054 (1.146-3.682)	0.016		
Nerve-Sparing	2.193 (1.204-3.993)	0.010		
PLND (yes/no)	2.477(1.442-4.255)	0.001		
Urination control function recovery				
Surgical technique (PPLRP/Intra-LRP)	0.169 (0.057-0.505)	0.001	0.169 (0.057-0.505)	0.001

CI, confidence interval; IIEF-5, Index of Erectile Function-5; Intra-LRP, intrafascial laparoscopic radical prostatectomy; NCCN, National Comprehensive Cancer Network; OR, odds ratio; PLND, pelvic lymph node dissection; PPLRP, pubovesical complex preserving technique for intrafascial laparoscopic radical prostatectomy; PSA, prostate-specific antigen.

### Comparison of sexual function recovery between PPLRP and intra-LRP groups

Both groups of patients had a history of fertility and subjectively considered that their sexual function was normal prior to the surgical intervention. Preoperative IIEF-5 scores were comparable between the two groups (p = 0.978)(Table 1). Conversely, postoperative IIEF-5 scores were significantly higher in the PPLRP group compared to the intra-LRP group (p < 0.001). Subjective recovery of sexual function to preoperative levels was 43.2% and 32.9% in the PPLRP and intra-LRP groups, respectively (p = 0.061), with 31.0% of patients in the PPLRP group and 19.4% in the intra-LRP group achieving satisfactory erection for sexual intercourse (p = 0.018) (Table 3). Multivariate analysis uncovered that age (p = 0.05) and surgical technique (p = 0.042) were independent predictors of postoperative sexual function recovery (Table 4).

## Discussion

The PPLRP technique is a modification of the Retzius-sparing [9–11] and the “Aphrodite’s veil” sparing [2,12,13] techniques used in RALP in the context of conventional laparoscopy. It leverages the 3–4 × magnification of conventional laparoscopy to facilitate dissection of fascial layers using a suction device for the blunt dissection of the “Aphrodite’s veil” from the prostatic capsule (Fig 1B) and for dissecting the avascular plane between the anterior surface of the prostate and the DVC (Fig 1C). Of note, this approach enales the preservation of all PVC structures, including surrounding the prostate (360°), the puboprostatic ligament, DVC, lateral fascial layers, NVB, and accessory pudendal artery. More importantly, preserving the anatomical support mechanisms of the prostate and the urinary tract facilitates early recovery of urinary control and sexual function postoperatively without compromising the PSM rate and oncological outcomes.

Noteworhily, no significant difference was noted in the PSM rate between the PPLRP and the conventional Intra-LRP groups (17.4% vs. 14.8%) and was comparable to the PSM rate associated with RALP (12.8%−19.2%) [4,28]. This may be ascribed to appropriate patient selection prior to the implementation of the PPLRP technique. ① Given that the dissection plane of the Veil-sparing technique is closer to the prostatic capsule than the conventional NVB, adherence to indications for nerve-sparing techniques [29] was ensured via multidisciplinary discussions. ② The 3–4 magnification provided by laparoscopy was leveraged to expose and dissect the fibromuscular tissue surrounding the prostatic apex and the posterior urethra (Fig 1C) to limit apical PSM. It is worthwhile emphasizing that the apical PSM rate was similar between the PPLRP and conventional Intra-LRP groups (6.5% vs 5.2%) and that reported for RALP (5.8%−5.3% [4,28]). Meanwhile, the 10-year bRFS (78.2% vs. 79.4%), CSS (94.3% vs. 94.2%), and OS (70.2% vs. 90.1%) rates were comparable between the two groups (p > 0.05). More importantly, these rates were consistent with the 10-year bRFS, CSS, and OS rates of 81%−88% [4,30], 97% [31], and 96.1%−79.5% [3] for RALP. Overall, these results collectively demonstrate that the application of PPLRP in selective patients does not affect the PSM rate and oncological outcomes.

Furthermore, compared with traditional Intra-LRP, PPLRP resulted in marked improvements in early postoperative urinary control, with control rates increasing from 61.9% to 78.1% at 24 hours after catheter removal and from 86.5% to 94.8% at 1 month. The outcomes were similar to previously reported rates for RALP (control rate at 1 week and 1 month: 20%−85% and 59%−92%, respectively) [9,10]). This may be attributed to the PPLRP technique maximizing the preservation of the anatomical structures around the prostatic urethra, thereby minimizing injury to the sphincters and supportive structures at the apex of the prostate. ① By avoiding the opening of the bilateral pelvic fascia and stripping the puborectalis muscle, the puborectalis muscle fibers adhere to the pelvic side fascia, avoiding excessive dissection.② The complex structure of the interweaving fibers formed by the aponeurotic region of the levator ani muscle and the striated muscle sphincter, which contains small nerve branches of the pudendal nerve [12,32], was preserved. ③ Dissection between the ventral prostate and the DVC in the avascular plane beneath the puboprostatic ligament allowed for the identification of the DVC complex and the small arteries traversing the Santorini plexus [9].④ Routine suture ligation of the DVC was avoided. In certain cases, excessively deep suture ligation may compromise the vascular supply of the urethra and the sphincter complex. Besides, in cases of perioperative DVC injury, hemostasis was generally achieved without ligation by applying direct pressure using a suction device against the superior pubic ramus and rapidly dissecting the DVC and its ventral connection to the prostatic urethra. In rare cases, suture ligation of DVC on the medial side was required.

As from the 6-month follow-up postoperatively, with a median follow-up duration of 45 months, 43.2% of patients in the PPLRP group subjectively reported that their sexual function had recovered to preoperative levels, with 31.0% of patients able to engage in sexual intercourse. As anticipated, postoperative IIEF-5 scores were significantly higher in the PPLRP group compared to the conventional Intra-LRP group. At the same time, it was also comparable to outcomes following RALP, where erectile function recovery rates ranged between 21%−44% at 6 weeks postoperatively, between 35%−57% at 3 months [28], and between 10%−50% at 12 months postoperative [4]. This outcome may be related to the technique not only partially retaining the lateral pelvic fascia and rarely damaging the NVB but also preserving the branches of the inferior hypogastric artery and other anatomical structures.

Urinary control and sexual function recovery following radical prostatectomy is an intricate process involving numerous factors such as patients’ conditions and surgical techniques. Herein, multivariate analyses unveiled that surgical technique was the sole independent factor for postoperative urinary function recovery, whilst surgical technique and age were identified as independent factors affecting sexual function, in line with previous reports for RALP [4,9,32,33]. Consequently, further studies are warranted to identify factors affecting early functional recovery associated with the PPLRP technique.

Nevertheless, several aspects necessitate attention during the implementation of PPLRP: ① To begin, compared with RALP, it is challenging to distinguish the anatomical plane between the base of the prostate and the bladder neck. Based on our experience, this challenge can be overcome by leveraging the 3-4x magnification and the enhanced visual field provided by conventional laparoscopy. In addition, the dissection should be initiated with a superficial incision at the junction between the bladder neck and the prostate in the 10−2 o’clock direction, and the bladder-urethral muscle fibers located at the junction area from the bladder neck to the prostate should be identified. With cephalad traction of the bladder, the junction area between the sphincter muscle located on the midline and the lateral sides should be meticulously dissected to preserve the bladder neck (Fig 1A).② During the initial application of the technique, it is recommended to select patients with medium-sized prostate and subsequently expand the indications for PPLRP following sufficient proficiency in the technique. The PPLRP technique preserves the PVC, and it is challenging to identify and dissect the avascular plane between the PVC and the prostatic urethra. Based on our experience, in patients with small prostates, the avascular plane at the junction of the PVC and the prostatic urethra is short, increasing the risk of damage to the urethral sphincter and the DVC perioperatively. In contrast, in patients with large prostates, the detrusor apron and the DVC are thinned and dispersed due to compression, thereby posing challenges in anterior dissection. ③ Preservation of the PVC, in conjunction with extensive preservation of the NVB, could lead to a narrow surgical field and a higher risk of intraoperative bleeding. The PVC (i.e., HOOD cover [17]) can be lifted using an aspirator to expose the surgical field (Fig 1D and E), and hemostasis can be achieved by securing small vessels and pedicles using titanium or Hem-o-lok clips, allowing the NVB to remain in close proximity to the subfascial layer and mitigating the risk of damage. Meanwhile, cranial traction of the mobilized prostate to fully expose the junction of the prostatic apex and the urethra enables maximal preservation of urethral length. ④ With the introduction of 4K/8K ultra-high definition and three-dimensional laparoscopic systems [34], as well as the progressive accumulation of surgical experience and technical improvements, LRP is capable of refined and precise anatomical dissection and achieved favorable curative effects comparable to those achieved with RALP. For the vast majority of hospitals without robotic equipment, this technique offers an affordable surgical alternative.

### Limitations of the study

The present study was retrospective in nature, and despite the use of propensity score matching to minimize confounding factors, randomized controlled trials remain the gold standard for evaluating surgical outcomes. Secondly, the results were derived from a single medical center with experienced physicians, limiting the generalizability of the findings to other institutions. Thirdly, the follow-up period was insufficient (median follow-up time 45–47 months), and the limited sample size may pose challenges in establishing definitive conclusions regarding outcomes such as overall survival, especially given the poor overall survival noted in the intra-LRP group. Considering that this technique does not require expensive robotic equipment and consumables, it can be implemented in medical institutions equipped with conventional laparoscopes. Nevertheless, future large-scale randomized trials with extended follow-up are warranted to validate the efficacy, safety, and cost-effectiveness of the PPLRP technique.

## Conclusion

The PPLRP technique is a laparoscopic modification of the Retzius-sparing and Veil-preserving techniques developed for robot-assisted surgery. Compared with the traditional intra-LRP technique, the PPLRP approach significantly improves early functional recovery without affecting PSM rates and oncological outcomes. Overall, it can be readily implemented in hospitals equipped with conventional laparoscopic systems.

## Supporting information

S1 Fig Intraoperative view of PPLRP (bilateral nerve-sparing). (PDF)

S1 Appendix Medical Ethics. (DOCX)
