# Supplementary material for: Application and efficacy of the pubovesical complex-preserving technique in intrafascial laparoscopic radical prostatectomy: A propensity score-matched analysis
Source: PLoS One. 2026 Mar 6;21(3):e0342248. doi: 10.1371/journal.pone.0342248 (PMC12965577; doi:10.1371/journal.pone.0342248)
Supplement: S1 Fig — (PDF) [file pone.0342248.s001.pdf]

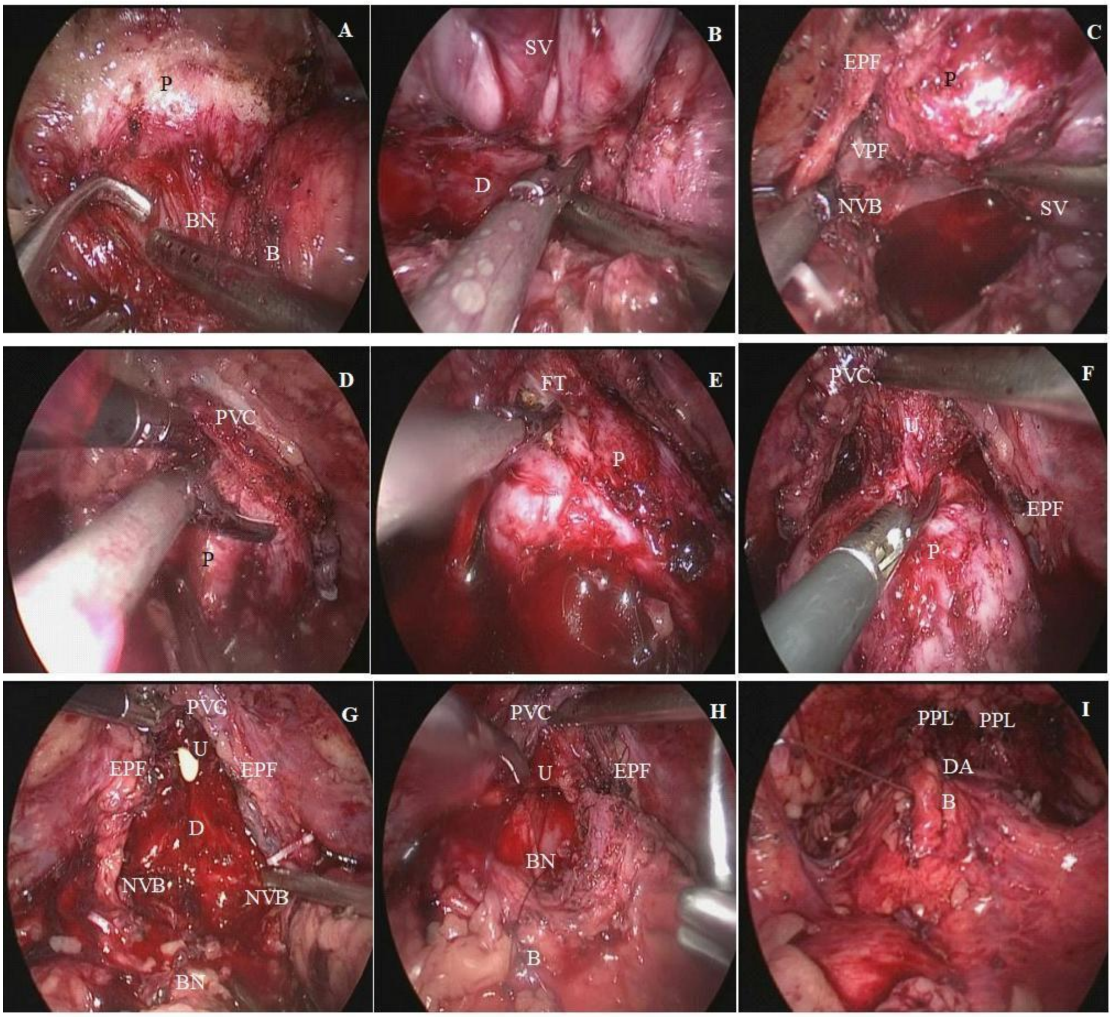


P=prostate,BN=Bladder neck,B=bladder,SV=seminal vesicle,EPF=endopelvic fascia,VPF=visceral prostatic fascia,NVB=neurovascular bundle,PVC=Pubovesical complex,FT=fibromuscular tissue,D=Denonvilliers'fascia,U= urethra, PPL=Puboprostatic ligament,DA = detrusor apron.

**S1_Figure A：Intraoperative view of PPLRP (bilateral nerve-sparing).**

The PPLRP technique was performed via an anterior approach. Firstly, the subcutaneous fat tissue over the prostate surface was removed, following which the bladder neck was transversely incised between the 10 to 2 o'clock positions at the junction of the bladder neck and prostate. Next, the bladder was retracted cephalad to expose and transect the bladder-urethral muscle fibers located at the junction, thereby preserving the bladder neck **(Fig. A).** The anterior and posterior lips of the bladder neck were divided using a harmonic scalpel or cold scissors. Afterward, the epididymis and vas deferens were exposed, and the Denonvillier's fascia was incised sharply or bluntly close to the epididymis **(Fig. B)**. Then, the dissection was extended bilaterally to release the NVB and free the pelvic side fascia **(Fig. C).**

Thereafter, the apex was dissected, and the DVC was detached from the prostate **(Fig. D)**. The fibromuscular tissue between the DVC and the prostate was dissected using cold scissors along the midline, and the urethra was transected **(Fig. E-F)**. At this stage, the entire PVC [including all structures surrounding the membranous urethra around the prostate ($360^0$), such as the puboprostatic ligament, DVC, detrusor apron, pelvic fascia, and NVB, etc.] was completely preserved **(Fig. G).** Following this, the posterior Denonvilliers' fascia was repaired. Anastomosis of the bladder neck and the membranous urethra was performed **(Fig. H),** followed by anatomical reduction of the PVC and closure of the ventral bladder neck incision **(Fig. I)**.

**Abbreviations:** PPLRP： pubovesical complex preserving technique in intrafascial laparoscopic radical prostatectomy

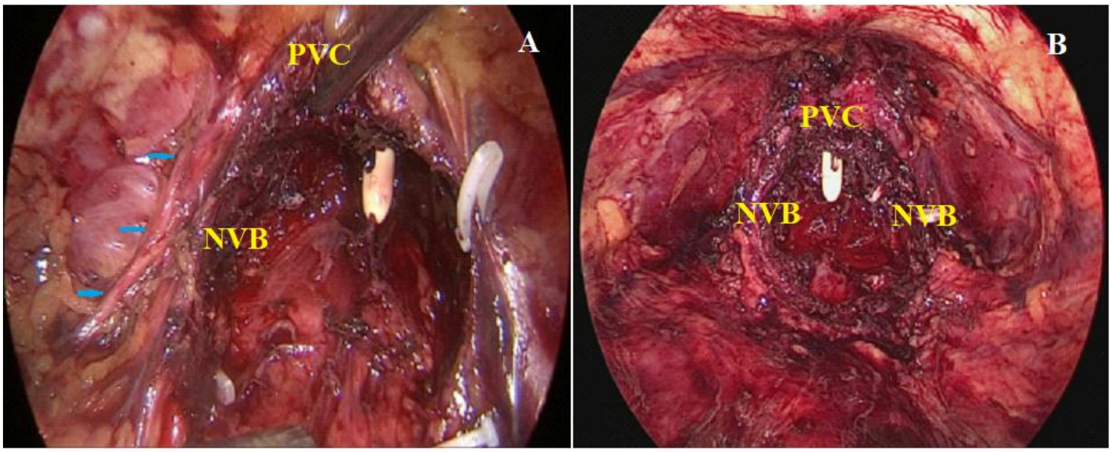


**S1_Figure B：4K ultra high definition conventional laparoscopy PPLRP technique.**

left nerve-sparing (A), bilateral nerves-sparing (B).Blue arrow : Accessory pudendal artery

**Abbreviations:** PPLRP：pubovesical complex preserving technique in intrafascial laparoscopic radical prostatectomy
