# Supplementary material for: Application and efficacy of the pubovesical complex-preserving technique in intrafascial laparoscopic radical prostatectomy: A propensity score-matched analysis
Source: PLoS One. 2026 Mar 6;21(3):e0342248. doi: 10.1371/journal.pone.0342248 (PMC12965577; doi:10.1371/journal.pone.0342248)
Supplement: S1 Appendix — (DOCX) [file pone.0342248.s002.docx]

Medical IEC-AF/17-1. 1

**Ethical review approval document**

Ethics review number: (Research) 2021-Ethics review-187

| Dealing time | 2021.8.6 | |
| --- | --- | --- |
| Review date | 2021.11.9 | |
| Scheme name | Research and application of the three-dimensional anatomical structure of the peripheral pelvic nerve network of the prostate | |
| Case number | (Research)2021-187-001 | |
| Department/Principal Investigator | Urology/Zhu Zengsheng | |
| Ethics review method | 口 Review of the meeting ☑ Quick review | |
| Review document | 1. Ethics Review Application Form  2. Feasibility Report of Zhejiang Provincial Medical and Health Research Project  3. Informed Consent Form (1.0/2021.8.1) | |
| Number of attending members: 16 members were expected to attend, 12 members actually attended, and 0 members abstained. | | |
| Voting results: 12 votes in favor; 0 votes in favor with amendments; 0 votes for reconsideration with amendments; 0 votes against. | | |
| Track review time | □3 months □6 months ☑12 months □Other | |
| Review comments | In accordance with the requirements of the National Health and Family Planning Commission's "Ethical Review Methods for Human Biomedical Research" (2016), the World Medical Association's "Declaration of Helsinki" (2013), and other regulations.  Precautions:  This research is effective for implementation within 3 years from the date of signing the document: if it is not implemented beyond the expiration date, this document will be invalid;  No modification may be made to any material without the review and approval of the committee;  If any serious adverse events occur during the research, please notify within 24 hours of knowing. | |
|  | The Ethics Committee;  In case of any deviation from the study protocol during the research process, a report of protocol deviation should be submitted in a timely manner:  The clinical study should be suspended or terminated in advance, and a suspension/termination review application should be submitted in a timely manner: The composition, duties, and operational procedures of this Ethics Committee shall comply with relevant laws and regulations.  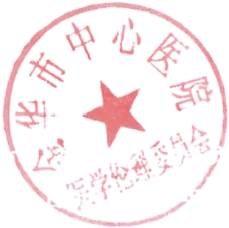Opinion of the Chairman: ☑Agree to carry out the scientific research project  Signature of Chairman/Deputy Chairman: 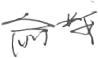  Yu Shi-an  The Medical Ethics Committee of Jinhua Central Hospital  2021-11-10 | ) |


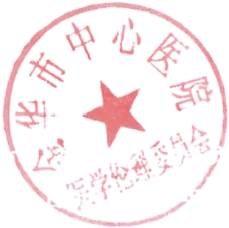
Ethics Committee Address: No. 365, Renmin East Road, Jinhua City, Zhejiang Province Contact: 0579-82556396
